# Supplementary material for: A Global Screen for Assembly State Changes of the Mitotic Proteome by SEC-SWATH-MS
Source: Cell Syst. 2020 Feb 26;10(2):133–155.e6. doi: 10.1016/j.cels.2020.01.001 (PMC7042714; doi:10.1016/j.cels.2020.01.001)

Q460N5 | PAR14_HUMAN | PARP14 BAL2 KIAA1268
Monomer MW [kDa]: 202.8 Monomer expected elution fraction: 34

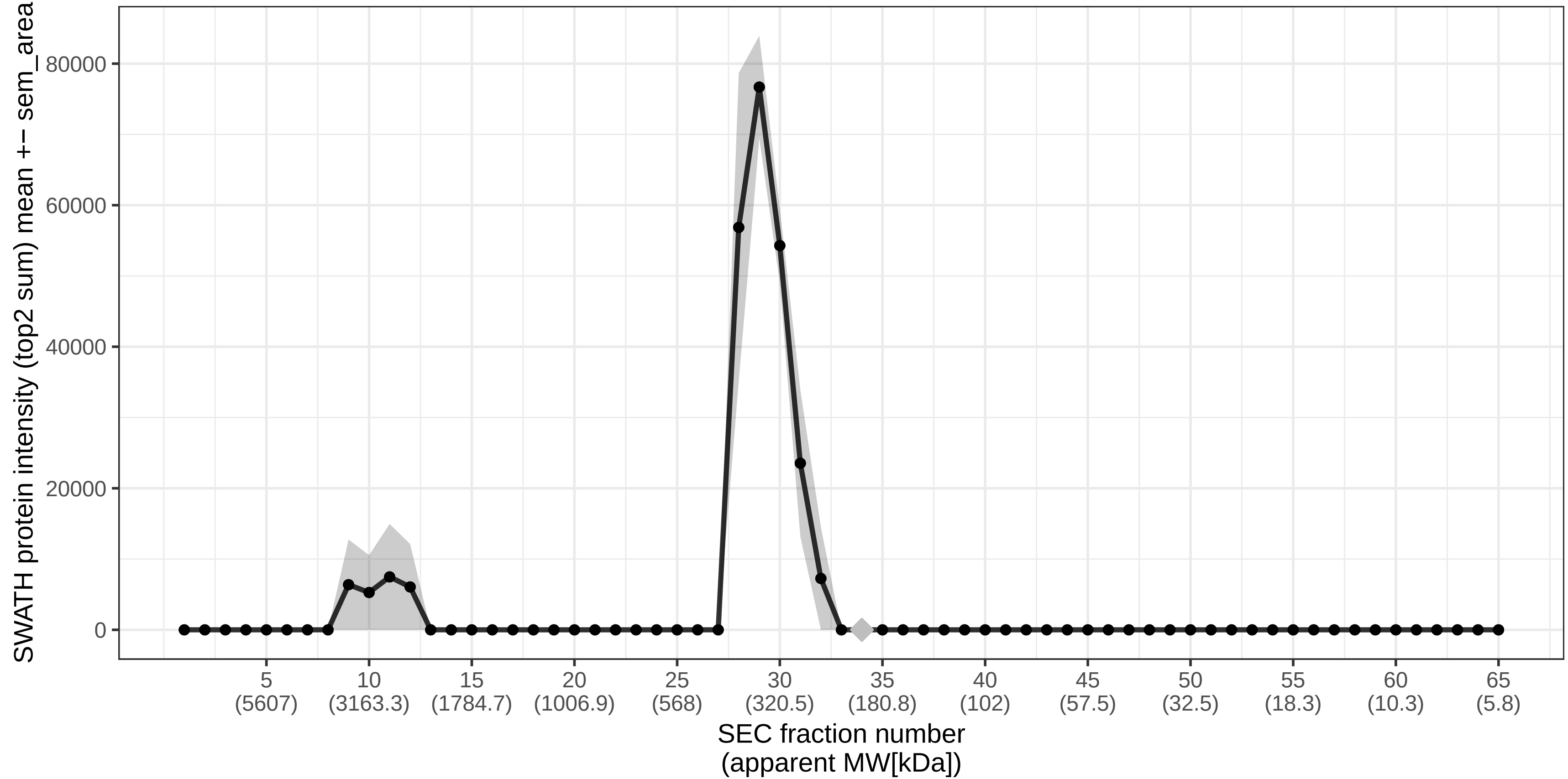

Supplement: Data S1. SEC-SWATH-MS Protein Chromatograms, Related to Figure 1 [file mmc6.zip › SECchrom_Q460N5_PAR14_HUMAN_PARP14_BAL2_KIAA1268.pdf]
